# Pathogenic potential and phylogenomic analysis of stx2-carrying O26:H11 Shiga toxin-producing Escherichia coli isolated from dairy products in France (2014–2024)

**DOI:** 10.1099/mgen.0.001647

**Published:** 2026-02-19

**Authors:** Nathan Soleau, Maxime Bruto, Sarah Ganet, Stéphane Bonacorsi, Aurélie Cointe, Delphine Sergentet

**Affiliations:** 1 Laboratoire d’Écologie Microbienne, Research group « Opportunistic Bacterial Pathogens and Environment », UMR5557, Université Claude Bernard Lyon I, VetAgro Sup, Marcy-l’Étoile, France; 2 Laboratoire d’Étude des Microorganismes Alimentaires Pathogènes–French National Reference Laboratory for Escherichia coli Including STEC (NRL-STEC), VetAgro Sup–Campus Vétérinaire, Université de Lyon, Marcy-l’Étoile, France; 3 UMR Mycoplasmoses animales, Université de Lyon, ANSES, VetAgro Sup, Lyon, France; 4 Service de Microbiologie, Centre National de Référence Escherichia coli, AP-HP, Hôpital Robert-Debré, Université Paris-Cité, IAME, UMR 1137, INSERM, 75018 Paris, France

**Keywords:** Bayesian inference, phylogenomics, raw milk products, O26:H11, Shiga toxin-producing *Escherichia coli* (STEC), *stx2*

## Abstract

Shiga toxin-producing *Escherichia coli* (STEC) are major foodborne pathogens causing sporadic enteric disease and paediatric haemolytic uraemic syndrome (HUS) cases globally. In France, STEC O26:H11 strains carrying the *stx2* gene have become the leading cause of paediatric HUS over the last decade. Concurrently, data from the French National Reference Laboratory show an increased proportion of *stx2*-carrying O26:H11 strains isolated from food, including raw milk products (RMPs). The consumption of RMP contaminated by this specific genotype has been linked to three out of four nationwide outbreaks between 2015 and 2024, confirming its emergence in France with RMPs as a potential source of infections. This study aimed to investigate the population structure, pathogenic potential and evolutionary dynamics of *stx2*-carrying O26:H11 STEC strains found in RMP in France. Using Illumina whole-genome sequencing, we (i) conducted a comparative analysis of human clinical strains and RMP isolates based on a set of accessory genes to assess pathogenic potential, (ii) performed phylogenomic analysis and (iii) explored evolutionary dynamics of major *stx2*-carrying STEC clonal lineages identified in RMPs using Bayesian evolutionary analysis sampling trees. Our results identified a predominant *stx2*-carrying O26:H11 clonal lineage, ST21-cl5, which became predominant in the raw milk production sector across multiple French regions and showed moderate diversification with evolutionary rates consistent with previously reported rates for STEC (i.e. 4.496×10^−7^ substitution/site/year). ST21-cl5 isolates from RMP displayed a high pathogenic potential, clustering closely with HUS-associated human isolates in both accessory genetic feature-based and core genome-based analyses. These findings suggest that clinical and RMP ST21-cl5 isolates evolved under similar selective pressures and shared a common ecological niche. Conversely, *stx2d*-carrying O26:H11 isolates also responsible for human infections in France might stem from a different source, as no *stx2d*-carrying strain was found among RMP isolates.

**Impact Statement**

Shiga toxin-producing *Escherichia coli* (STEC) are major foodborne pathogens responsible for life-threatening infections, especially in young children. Since the mid-2010s, strains of serotype O26:H11 carrying the *stx2* gene subtype have become the first leading cause of paediatric haemolytic and uremic syndrome in France. This emerging genotype in humans has also been increasingly found in food samples by the French National Reference Laboratory for *E. coli*, with raw milk product (RMP) at the forefront of this phenomenon. This study thus provides a first insight into the population structure and evolutionary dynamics of such STEC strains within the French raw milk production sector and how they relate to human clinical strains over the last decade. Here, we untangle the role of the raw milk production sector in the transmission of *stx2*-carrying O26:H11 STEC to humans and demonstrate that, while RMP seems to be largely involved in the transmission of strains from a particular clonal lineage (ST21-cl5) of this emerging genotype to humans, some strains from other clonal lineages (e.g. the ‘new French clone’) found in human cases could not be linked to RMPs, suggesting the existence of other transmission routes.

## Data Summary

All *Escherichia coli* genome sequences used in this study are available from the National Center for Biotechnology Information Sequence Read Archive under BioProject PRJNA1244818. Their associated accession numbers (SAMN47731773 to SAMN47731902), metadata and *in silico* typing information are available in Table S1 (available in the online Supplementary Material).

## Introduction

Shiga toxin-producing *Escherichia coli* (STEC) are foodborne zoonotic pathogens that are frequently responsible for large-scale outbreaks with strong public health consequences. The clinical manifestations of a STEC infection range from self-limiting diarrhoea to severe complications such as haemolytic uraemic syndrome (HUS), which primarily affects young children and immunocompromised individuals [1]. The pathogenicity of these bacteria is largely due to their *stx* gene, carried by a prophage integrated into their chromosome and coding for a Shiga-toxin [2]. Thus far, 2 types of the *stx* gene have been described: *stx1*, with 4 subtypes (*stx1a*, *stx1c*, *stx1d* and *stx1e*), and *stx2*, with 12 subtypes (*stx2a* to *stx2l*) [3]. Highly pathogenic STEC strains also generally possess the *eae* virulence gene, which encodes intimin – a membrane protein involved in the colonization of the gastrointestinal tract with the formation of characteristic ‘attachment and effacement’ lesions [4].

While microbiologists have described hundreds of STEC serotypes, only a small number are responsible for most of the severe human infections. In France, six predominant serotypes are involved in both sporadic human cases and collective outbreaks: O26:H11, O80:H2, O103:H2, O111:H8, O145:H28 and O157:H7 [5,6]. For several decades, serotype O157:H7 was associated with the highest number of human STEC infections and subsequent HUS cases. However, since the mid-2010s, French paediatric nephrology services have observed a sharp increase in HUS cases linked to O26:H11 and O80:H2 infections, coupled with a significant decrease in cases linked to serotype O157:H7 [6,7]. Similar to observed trends in other European countries [8], strains of serotype O26:H11 have become the leading cause of paediatric HUS in France.

A subset of STEC strains, particularly those harbouring the *stx2*, has been strongly associated with an increased risk of developing paediatric HUS and other severe symptoms compared to *stx1*-positive strains [9,10]. In light of the growing concern surrounding the pathogenicity of such subtypes, the French Agency for Food, Environmental and Occupational Health and Safety (ANSES) recently revised its criteria for defining pathogenic STEC, shifting from a classification based on serotype-virulence factor associations to one primarily focused on the presence of specific virulence genes, notably *stx2a* and *stx2d* [4,11].

Cattle have been identified as asymptomatic carriers of STEC, including O26:H11, in their gastrointestinal tracts and serve as the primary reservoir for this pathogen. STEC contamination of foodstuffs can occur at various stages along the food chain, with milking and slaughtering being the most sensitive stages. Hence, poor hygiene practices during these processes can greatly increase contamination risks [12]. Although STEC can be found in a wide range of food products, in France, food-related human infections have mostly been linked to the consumption of undercooked ground meat, raw milk and its derivatives [6,13].

In parallel with the rising number of paediatric HUS cases linked to serotype O26:H11, internal data from the French National Reference Laboratory for STEC (FNRL-STEC) show an increase in the proportion of *stx2*-carrying O26:H11 STEC strains among all O26:H11 strains isolated from food matrices (Fig. S1). Between 2010 and 2016, these strains represented an average of 3.93% of all O26:11 isolates, rising to 24.03% during the period 2016–2023. Food business operators from the French raw milk production sector also report an increase of STEC O26:H11 isolates carrying the *stx2* gene in environmental or food samples collected as part of their microbiological self-monitoring plans [14], and three out of four major O26:H11 nationwide outbreaks were linked to the consumption of raw milk products (RMPs) [15,18]. Compared to other European countries, France has a long tradition of raw milk cheese production and currently has 46 cheeses under protected designation of origin (PDO), 77.2% of which are still required to be made with raw milk and with bovine RMPs accounting for 87% of the total commercialized volumes in 2023 [19,20]. Hence, this previously rare genotype of STEC O26:H11 seems to have emerged in France with bovine RMPs as a potential major source of human infections and HUS in France.

In this context, this study aimed at giving an overview of the population of *stx2*-carrying O26:H11 STEC strains found in RMPs in France in terms of pathogenic potential to humans, phylogenetic structure and evolutionary dynamics. We thus (i) conducted a genomic comparative analysis of human clinical strains and RMP isolates based on a selection of accessory genes including virulence-associated genes (VAGs), antimicrobial resistance genes (ARGs) and plasmid replicons (PRs) to assess their pathogenic potential and (ii) performed a phylogenomic analysis to investigate the population structure of such strains. Finally, we (iii) explored the evolutionary dynamics of major *stx2*-carrying O26:H11 STEC clonal lineages found in the bovine RMP sector to provide insight into their emergence and diversification timelines.

## Methods

### Bacterial isolate selection and whole-genome sequencing

**Fig. 1.**
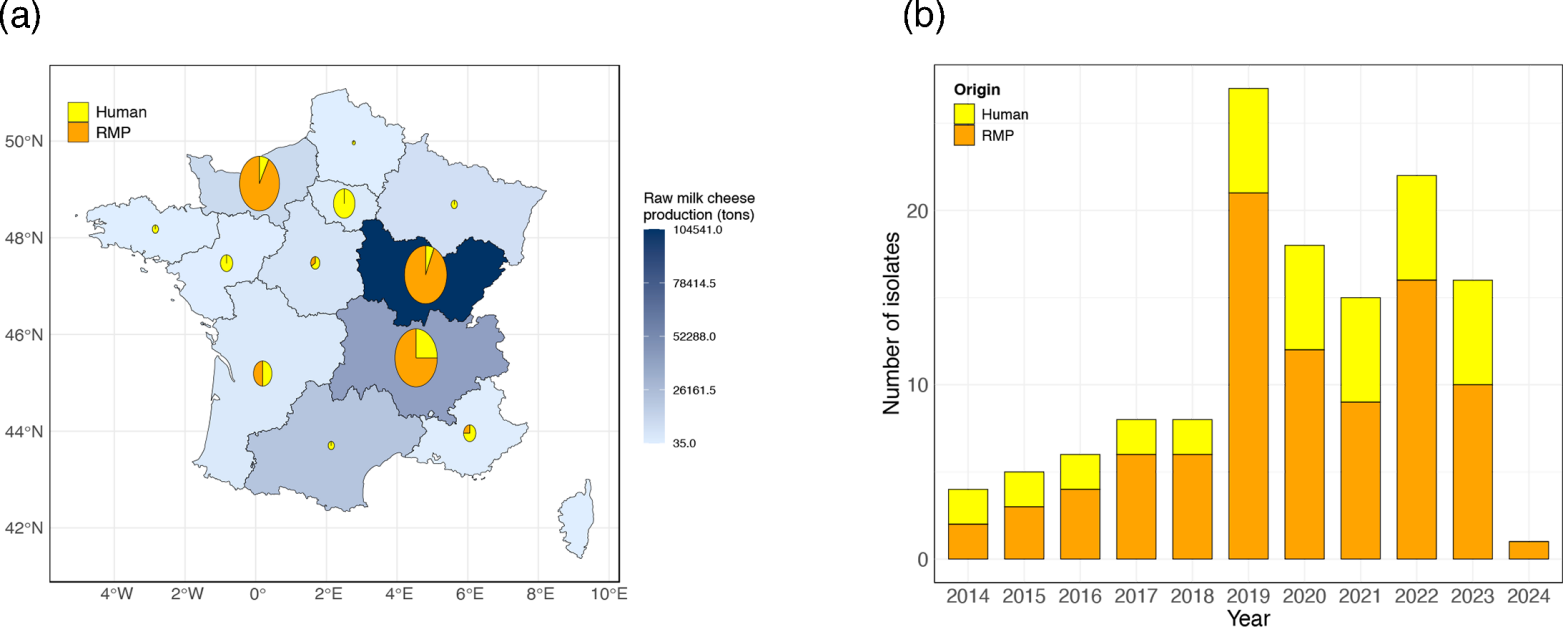
Geographical (a) and temporal (b) distributions of isolates included in this study.

STEC strains were collected as part of the routine microbiological testing and nationwide surveillance activities carried out by the FNRL for *E. coli*. We selected all *stx2*-carrying O26:H11 STEC isolates from bovine RMPs for which both geographical and temporal metadata were available. In total, 90 isolates, isolated between January 2014 and the end of January 2024 and originating from six French regions, were included in this study (Fig. 1). These 90 isolates represent 43.6% (90/206) of all *stx2*-carrying O26:H11 RMP isolates stored at the FNRL over the same period.

In addition, a selection of 40 *stx2*-carrying O26:H11 STEC strains isolated from clinical cases of STEC infections (36 with HUS and 4 with bloody diarrhoea) between 2014 and 2023 was provided by the microbiology unit of the University Hospital Robert-Debré (French National Reference Centre for *E. coli*) for genomic comparative analysis with the above-mentioned RMP isolates. A more detailed description of all included isolates is available in Table S1.

All 130 strains were subjected to genomic DNA extraction with the Promega® Wizard Genomic DNA purification kit according to the manufacturer’s instructions. Subsequently, DNA quality check and quantification were performed using a NanoDrop One™ microvolume spectrophotometer (Thermo Fisher Scientific, Waltham, MA, USA) and the Qubit™ dsDNA HS 1X kit (Thermo Fisher Scientific, Illkirch, France), respectively. Sequencing libraries were prepared from the genomic DNA using a Nextera sample preparation kit (Illumina, Inc., San Diego, CA, USA) and were sequenced with a 150 bp paired-end whole-genome sequencing strategy on an Illumina NovaSeq 6000 sequencing platform (Illumina, Inc.) by an external service provider (Novogene, Cambridge, UK).

### Virulome, resistome and PR characterization and comparative analysis of clinical and RMP isolates

Raw sequencing reads were first analysed with the STEC pipeline 1.0 from the Sciensano Galaxy online platform [21]. Briefly, reads were quality-checked and trimmed using the FastQC v 0.12.1 [22] and the Trimmomatic v 0.38 [23] tools, respectively. Genome assembly was performed with SPAdes v 3.13.0 [24], and assemblies were subsequently used for *in silico* characterization with multiple tools including SerotypeFinder [25], VirulenceFinder [26,27] and PlasmidFinder [28]. Antimicrobial resistance (AMR)-associated genes were detected with the ResFinder tool [29] available on the Center for Genomic Epidemiology (CGE) online platform (http://genepi.food.dtu.dk/resfinder). Finally, multilocus sequence types (MLST) were assigned according to the Warwick Medical School scheme [30,31].

Pathogenic potentials of *stx2*-carrying O26:H11 STEC strains from the French raw milk production sector were investigated through a comparative analysis of the virulome, resistome and PRs of RMP and clinical isolates. Similar to a previous study by Michelacci and colleagues [32] on a set of clinical isolates only in Italy, we performed a hierarchical clustering on principal components (HCPCs) [33] based on the detection of VAGs, PRs and ARGs. This approach combines both a multiple correspondence analysis and hierarchical clustering to first reduce the dimensionality of our dataset before applying a clustering method. Each cluster can then be interpreted based on the principal components, allowing the groups formed to be described according to the original characteristics of the data, herein the positive or negative association with a set of accessory genes. The objectives were to partition isolates into several distinct profiles based on virulence, AMR and PR (VAG-ARG-PR) and to assess the homogeneity or heterogeneity of each profile in terms of strain origin (RMP or clinical). In total, 120 qualitative variables were included in the HCPC analysis comprising 19 AMR-related genes, 82 VAGs and 19 PRs (Table S1). The multiple correspondence analysis and hierarchical clustering were performed with the R software v 4.4.1 [34] using the FactoMineR package [35], and to characterize the clusters, the v.test statistic was calculated for each gene, comparing the proportion of presence within the cluster to the global proportion [33,35, 36].

### SNP-based phylogenetic analysis of STEC isolates from human and RMP origin

Prior to the SNP-based phylogenetic analysis, the overall population structure of these isolates was broadly explored using a core genome MLST (cgMLST) approach based on the *E. coli* INNUENDO scheme implemented on the external ARIES Galaxy platform hosted by the Istituto Superiore di Sanità (European Union Reference Laboratory for STEC). Allele calling was performed with ChewBACCA v 2.0 [37], and the cgMLST tree was generated with the ChewTree tool v 1.0 and subsequently visualized and annotated also using Interactive Tree of Life (iTOL).

Then, to explore the intrinsic phylogenetic structure of the population of *stx2*-carrying O26:H11 STEC isolates from RMPs and their clonal relationships to human isolates, all 130 genomes (90 from RMP and 40 from human source) were mapped to the reference genome of the SH9 RMP isolate [National Center for Biotechnology Information (NCBI) SRA accession number: SAMN47731773], and an alignment of core SNPs outside of recombinant regions was generated with snippy v 4.6.0, snippy.core v 4.6.0 [38] and Gubbins v 3.2.1 [39]. The multiple sequence alignment (MSA) file of concatenated core SNPs was then used to construct a maximum-likelihood phylogenetic tree using IQ-TREE with a general time reversible (GTR) model of substitution with 3 categories for among-site rate heterogeneity (GTR+F+ASC+R3) and with 1,000 non-parametric bootstrap replicates [40,41]. The resulting tree was visualized, annotated and rooted midpoint on the iTOL [42] online platform. Additionally, a pairwise distance matrix of SNPs between STEC isolates was computed using snp.dist v 0.8.2 [43] (Table S2).

The reference genome SH9 was selected based on its high-quality assembly metrics and relatively early isolation date. To assess potential reference bias, we initially repeated the SNP analysis using two additional reference genomes with distinct cgMLST tree positions: SH64 and SH59 (intermediate position, Fig. S2). Across these analyses, we observed minor to no differences in the overall tree topology, with clade assignments and major branching patterns remaining stable. Since the three genomes tested as reference produced congruent topologies, only the tree based on the SH9 reference genome was retained and presented.

### Evolutionary dynamics of the ST21-cl5-RMP and ST29-cl3-RMP major clonal lineages

RMP genomes from the major circulating ST21-cl5 and ST29-cl3 clonal lineages were selected for further phylodynamic analysis to investigate their evolutionary history. For each clonal lineage, a second recombination-free phylogeny based on core SNPs and a maximum-likelihood tree with only the aforementioned isolates were constructed as previously described. The temporal signal in our heterochronous dataset was assessed with a root-to-tip regression analysis in TempEST v 1.5.3 with the ‘best-root’ option selected [44]. Correlation between genetic divergence and sampling dates, and dispersion around the regression line were evaluated based on the correlation coefficient and the *R*^2^ values. Upon verification of a positive correlation between genetic distances and sampling times, we proceeded with the construction of a timed phylogeny in Bayesian Evolutionary Analysis Sampling Trees 2 (BEAST2) v 2.7.7 [45]. The best-fitting tree model was determined by testing six model combinations with varying molecular clock and demographic priors: [strict molecular clock, optimized relaxed molecular clock] + [coalescent constant population model, coalescent exponential population model, Coalescent Bayesian Skyline model]. A GTR substitution model along with a discrete gamma site model with a category count of four was set for all tested combinations, and other priors were left as default. All Markov chain Monte Carlo generations were run with 10 million steps, and trees were sampled every 1,000 steps. Model comparison was performed using the nested sampling package v 1.2.2 [46] implemented in BEAST2 for marginal likelihood estimation and calculation of the Bayes factor and resulted in the selection of the strict molecular clock model associated with the coalescent constant population demographic model. Three additional independent analyses with the latter priors were conducted. LogCombiner was then used to combine all four runs with a 10% burn-in, and TreeAnnotator was used to generate a maximum clade credibility tree which was visualized and annotated with FigTree v 1.4.4. LogCombiner output was imported in Tracer v 1.7.2 [47] to estimate the tMRCA and the mean clock rate after evaluation of effective sample size values for key parameters (≥200) and of proper convergence. Finally, to assess *a posteriori* the strength of the temporal structure of our dataset, we performed a date randomization test with ten date-randomized replicates with our selected model as previously described by Duchene *et al*. [48] and Wang *et al*. [49].

## Results

### Comparative analysis of RMP and clinical O26:H11 STEC isolates

**Fig. 2.**
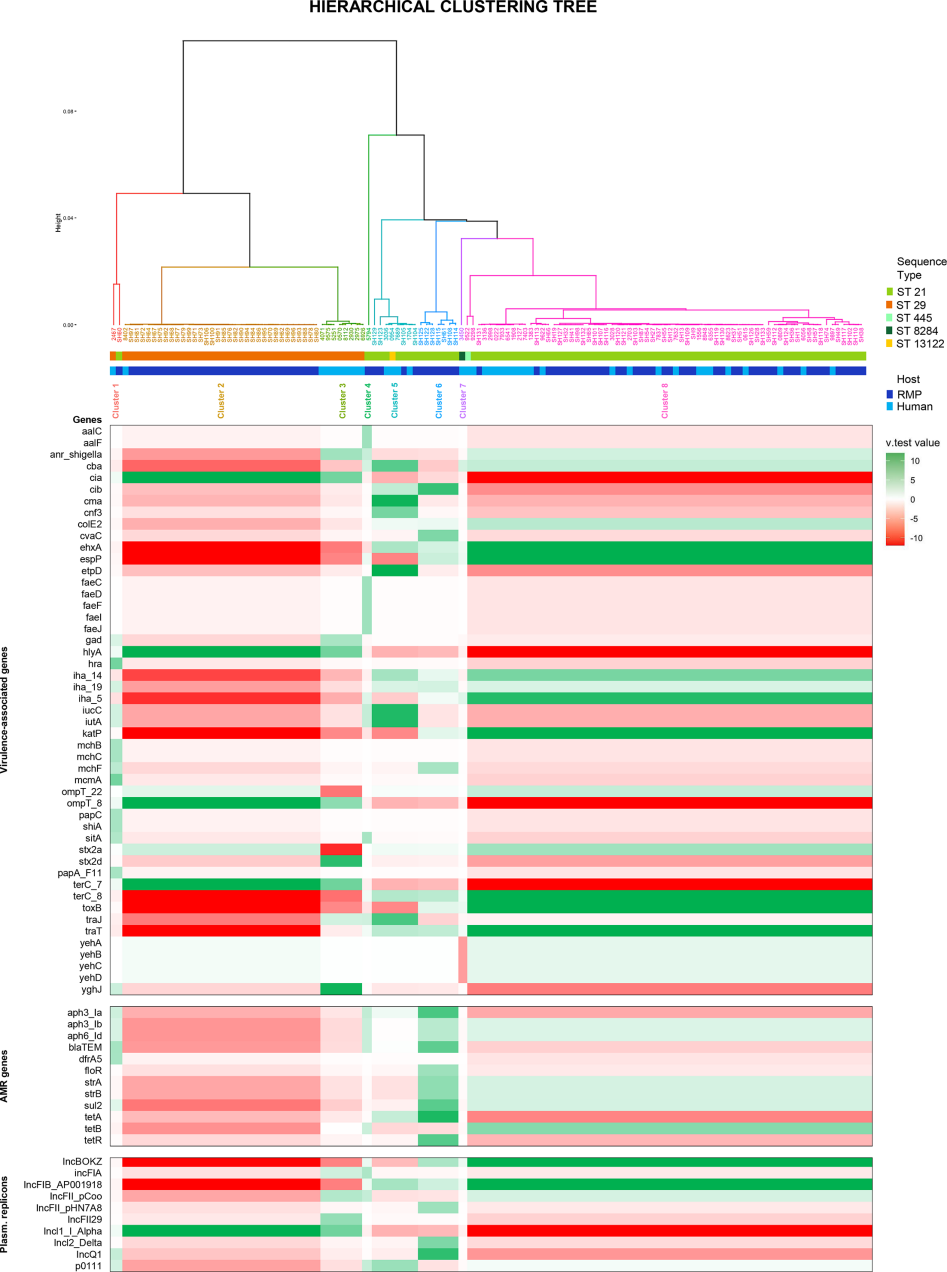
Hierarchical clustering tree resulting from the HCPC analysis displaying corresponding statistically significant associations of clusters with accessory genetic features. Positive and negative associations of accessory genes with clusters are indicated in green and red, respectively. The magnitude of the significance of the association is given by the v.test.value (|v.test|>1.96 corresponds to a *P*-value<0.05, suggesting statistical significance) [36]. Only genes showing a statistically significant association after false discovery rate correction (Benjamini–Hochberg procedure, *P*_adjusted<0.05) with at least one cluster are displayed.

Pathogenic potential of stx2-carrying O26:H11 STEC strains found in RMPs in France between 2014 and 2024 was assessed through a comparative analysis with human clinical isolates from the same period. In total, 90 strains from RMP originating from 6 French regions and 40 clinical isolates from the same period were included in this study. A HCPC analysis [33] was used to identify patterns of accessory genes, including VAGs, ARGs and PRs, which might differentiate or cluster together strains from different host sources based on their genomic features. Preliminary multiple component analysis based on these selected accessory genome features for both clinical isolates and RMP isolates showed that 59.21% of the variance could be attributed to ten dimensions. Hence, the first ten dimensions were kept for subsequent hierarchical clustering. The clustering process then revealed eight groups with significant differences in accessory gene composition. Five clusters grouped seven or more isolates, and the three others comprised only one or two isolates. Details regarding the associations between clusters and the presence/absence of considered genetic features are presented in Fig. 2 along with the partition of all isolates, and the complete presence/absence patterns are presented in Fig. S3. Notably, clusters were mostly characterized by the absence or by different combinations of virulence genes usually carried on a large pO26 virulence plasmid. Clusters 1 (C1), 2 (C2) and 3 (C3) were composed of 2, 34 and 8 isolates, respectively, and were all lacking the *ehxA*, *katP*, *espP*, *etpD* and *toxB* virulence genes and the plasmid conjugation-associated *traJ* and *traT* genes. However, isolates from human and RMP origin with this baseline genetic profile grouped separately in C2 (33 RMP isolates and only one human isolate) and C3 (8 human isolates only) based on their *stx2* gene subtype and the presence of the *yghJ* gene. Cluster 4 was composed of one clinical isolate which was mainly characterized by the presence of the *fae* operon associated with the production of fimbriae and by a positive association with ARGs *aph3_Ia*, *aph3_Ib* and *aph6_Id*. Cluster 5 included eight isolates from both human and RMP origin. Isolates from this cluster carried the following combination of pO26-related genes, *ehxA*+/*katP*-/*espP*-/*etpD*+/*toxB*- and other virulence factors such as the genes *iucC* and *iutA*, involved in iron acquisition. Cluster 6 grouped seven RMP-only isolates which displayed a more complete set of pO26 virulence genes (*ehxA*+/*katP*+/*espP*+/*etpD*-/*toxB*+) and were found to carry multiple ARGs mediating multi-drug resistance such as *aph3_Ia*, *aph3_Ib*, *strA/B*, *blaTEM*, *floR*, *strA/B*, *sul2* and *tetA/R*. Finally, cluster 8 included the most isolates and grouped together 24 human strains and 45 RMP strains. This VAG-ARG-PR profile was defined by the same pO26 gene combination as C6 but had a different set of ARGs with *tetB* instead of *tetA* and lacked the *aph3_Ia*, *blaTEM*, *floR* and *tetR* genes. The sole isolate from cluster 7 exhibited the same VAG-ARG-PR profile as isolates from C8 but was missing the *yeh* gene cluster responsible for the production of membrane proteins and adhesins.

### Population structure of *stx2*-carrying STEC O26:H11 isolated from RMPs and phylogenetic relationships with clinical isolates

**Fig. 3.**
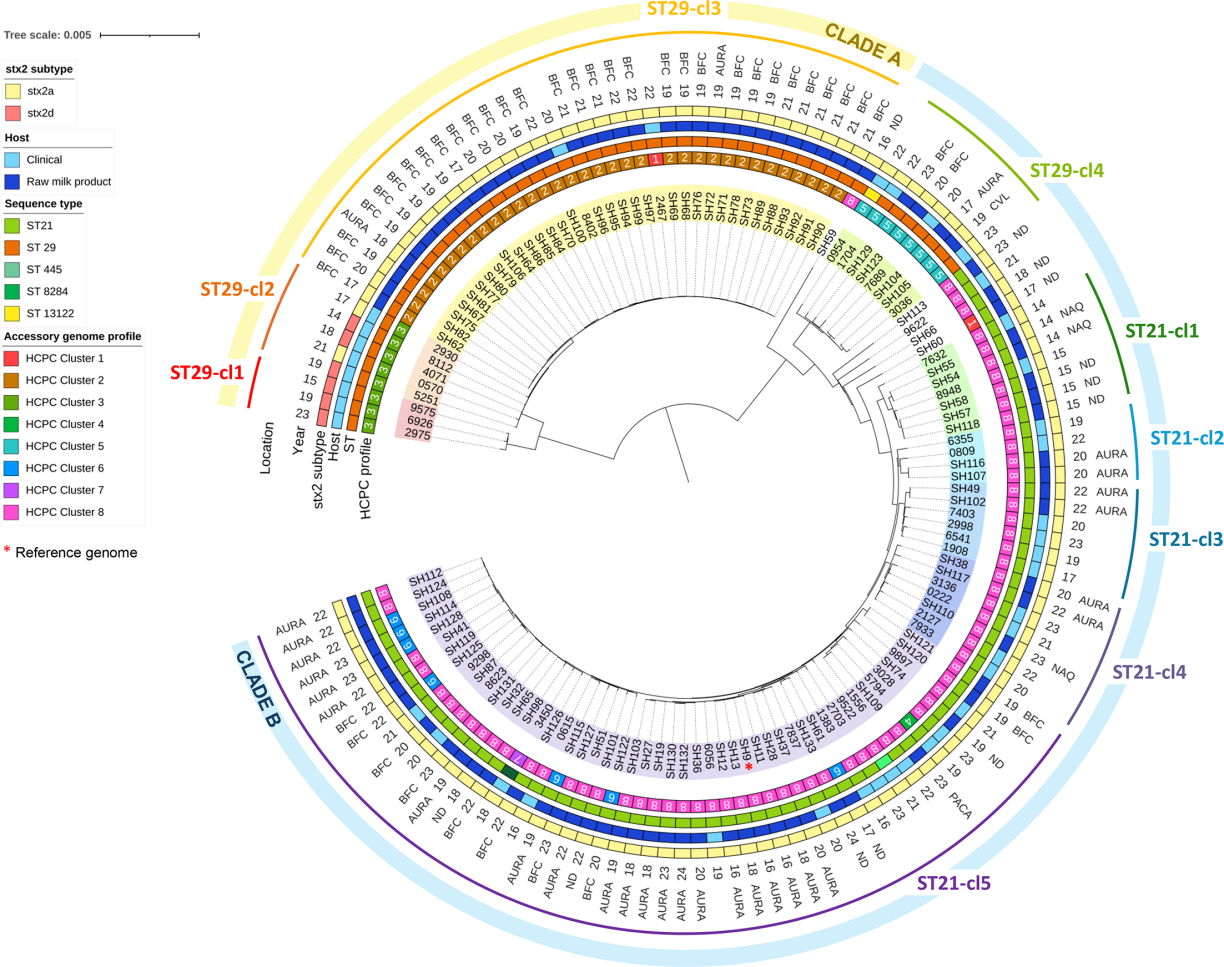
Core SNP-based maximum-likelihood phylogenetic tree. Isolate names from the same clonal lineage are shaded in the same colour. Metadata associated with location, year of isolation (e.g. 22 means 2022), stx2 gene subtype, host type, ST and HCPC profile are represented by coloured rings. Abbreviations for the regions of production for RMP and the regions of origin for clinical cases are the following: AURA, Auvergne–Rhône–Alpes; BFC, Bourgogne–Franche–Comté; ND, Normandie; NAQ, Nouvelle–Aquitaine; PACA, Provence–Alpes–Côte-d’Azur. The most outer rings describe the nine identified circulating clonal lineages and their corresponding clade. The reference genome used for the analysis is indicated with a red asterisk.

The genomic diversity and population structure of *stx2*-carrying STEC O26:H11 strains and their relatedness to clinical isolates were evaluated through a phylogenomic analysis. A core genome-based maximum-likelihood phylogenetic tree was generated with IQ-TREE after recombination filtration with Gubbins. In this study, 127 out of 130 isolates belonged to 2 major sequence types (STs), namely, ST21 (*n*=76) and ST29 (*n*=51), and interestingly, all RMP isolates carried the *stx2a* gene only, whereas human isolates had either *stx2a* or *stx2d*. Three other STs were detected, ST445 (*n*=1), ST8284 (*n*=1) and ST13122 (*n*=1), and corresponded to single allele variants of either one of ST21 and ST29. The overall population of both RMP and human isolates was genetically diverse with pairwise core SNP distances ranging from 0 to 2,817 with a mean distance of 1,184 SNPs. Two main groups of isolates were defined, clade A and clade B (Fig. 3), and were further subdivided into nine subclades, hereafter denominated clonal lineages, and five singletons. The overall population structure inferred from the cgMLST analysis (Fig. S2, Tables S3–S5) was highly consistent with that obtained from the core SNP phylogeny, indicating strong concordance between the two approaches. The inclusion of isolates within the same clonal lineages was stable across both analyses, supporting the robustness of the obtained core SNP phylogenetic clustering. In detail, ST21 strains were distributed into one major clonal lineage, ST21-cl5 (*n*=50), and four minor clonal lineages, ST21-cl1 to ST21-cl4 (*n*=4–7). Genomes from ST21-cl5 differed from 0 to 68 SNPs, with a mean and a median pairwise core SNP distance of 37.7 and 40 core SNPs, respectively (Fig. S4), and originated from both human (*n*=13) and RMP (n=37) sources and displayed various VAG-ARG-PR profiles with HCPC profile 8 being the major one. Notably, some RMP and human genomes were highly similar with a genetic distance under 10 SNPs and with closely spaced sampling dates (SH87 and 9298 for instance). RMP isolates from this clonal lineage were found in four different regions and over a 9-year period (2016–2024). Strains from clonal lineage ST21-cl1 were isolated in 2014 and 2015, and this lineage has not been detected in either RMPs or clinical cases since. Clonal lineages ST21-cl2, ST21-cl3 and ST21-cl4 comprised clinical and RMP genomes with sampling dates ranging from 2017 to 2023 and within-lineage SNP pairwise distances ranging from 0 to 87 SNPs. HCPC profile 8 was the only VAG-ARG-PR profile of these lineages, regardless of the isolates’ source host. Within clade B, all ST21 lineages described above also clustered with a ST29 lineage, ST29-cl4. On the other hand, clade A included ST29 isolates, and within this clade, human and RMP isolates tended to cluster separately, except for two human isolates in ST29-cl3. ST29-cl1 and ST29-cl2 grouped exclusively human isolates displaying HCPC profile 3, characterized in part by the *stx2d* gene, which caused HUS over the whole study period. Finally, the majority of ST29 isolates from RMPs came from the Bourgogne–Franche–Comté region and formed, along with two human isolates, a less diverse clonal lineage (i.e. ST29-cl3), with a mean pairwise distance of only 6.2 SNPs between genomes.

### Evolutionary history of the major ST21-cl5 and ST29-cl3 clonal lineages in the French raw milk production sector

**Fig. 4.**
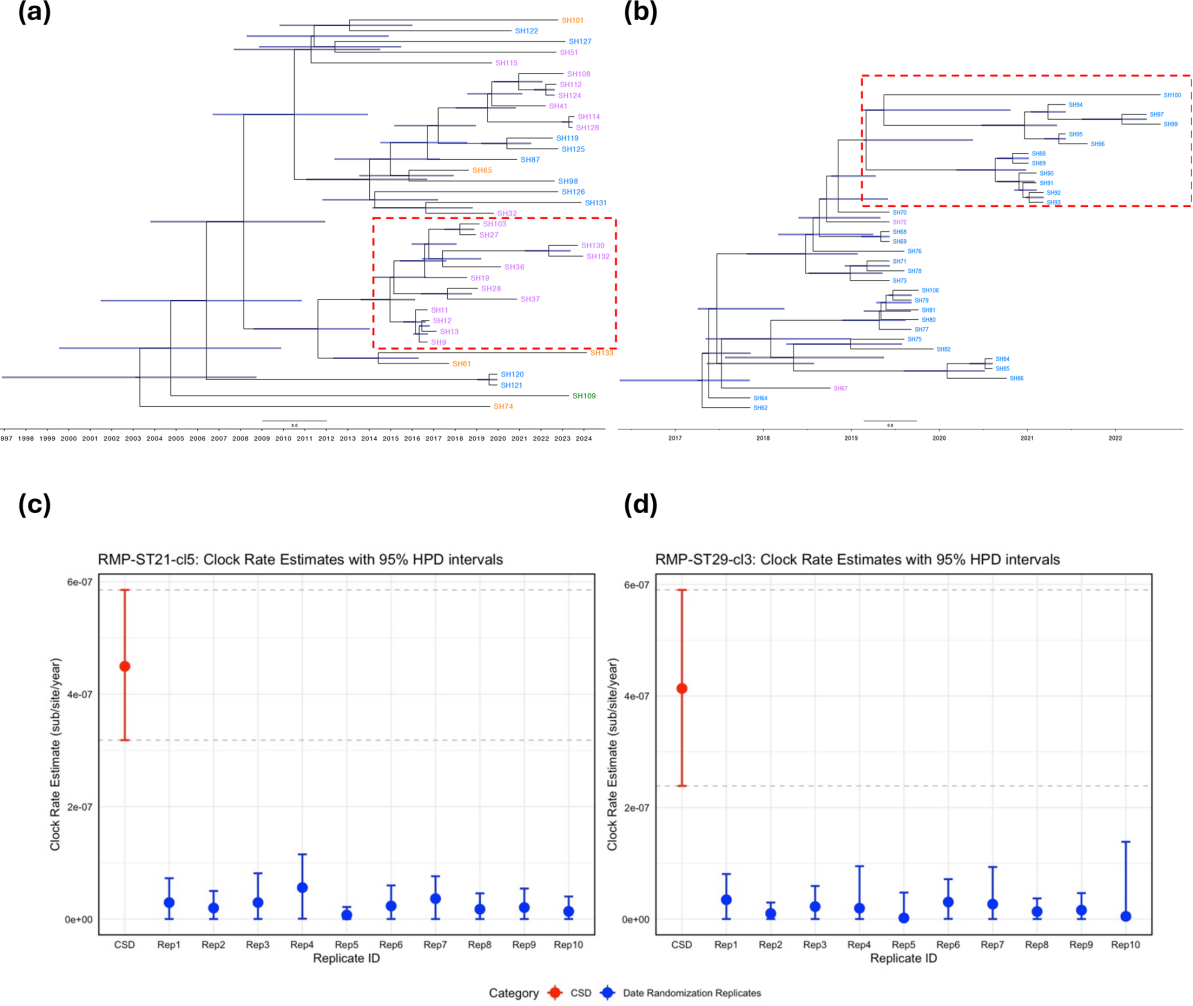
Maximum clade credibility timed trees of major *stx2*-carrying O26:H11 STEC clonal lineages found in RMP and clock rate estimates of date-randomized replicates after Bayesian analysis with their 95% HPD intervals. (a) RMP-ST21-cl5. (b) RMP-ST29-cl3. Isolates are coloured depending on their region of origin: orange, Normandie; blue, Bourgogne–Franche–Comté; pink, Auvergne–Rhône–Alpes; green, Provence–Alpes–Côte-d’Azur. Node bars represent the 95% HPD intervals for node heights. Clock rate estimates (red) and date randomization test with ten replicates (blue) with their 95% HPD intervals are presented: (c) RMP-ST21-cl5 and (d) RMP-ST29-cl3. CSD, correct sampling date.

We explored the evolutionary dynamics of the two dominant clonal lineages identified in our initial phylogenomic analysis within the RMP sector, specifically to provide a timeline for the emergence and diversification of these lineages in such a reservoir. Raw sequencing reads of RMP isolates from each lineage, namely, ST21-cl5 and ST29-cl3, were aligned to a lineage-specific reference genome (the isolate with the oldest isolation date), and core SNPs were identified and filtered to exclude recombinant regions, resulting in MSA of concatenated non-recombinant core SNPs. The obtained MSAs were used to generate core genome-based maximum-likelihood trees with IQ-TREE. After assessment with TempEST, RMP-ST21-cl5 and RMP-ST29-cl3 both had positive temporal signal with correlation coefficient values of 0.59 and 0.74, respectively. Bayesian inference of time-calibrated phylogenies was performed (Fig. 4a, b), and estimates for the times to the most recent common ancestor (tMRCA) and evolutionary rates are available in Table 1. *A posteriori* date randomization tests were performed for each lineage and indicated strong temporal structures within our datasets as none of the 95% highest posterior density intervals (HPD intervals) for clock rate estimates overlapped between the analyses with correct sampling dates and those from date-randomized replicates (Fig. 4c, d). Based on the overall structure of the inferred timed phylogenies, ST21-cl5 seemed to be more genetically heterogeneous than ST29-cl3, and its multiple clones broadly spread to several regions, except for one sublineage which was specific to the Auvergne–Rhône–Alpes region (framed in Fig. 4a). Conversely, for ST29-cl3, almost all isolates were restrained to the Bourgogne–Franche–Comté region, and a single sublineage that appeared around 2019 seems to have superseded all other ST29-cl3 clonal sublineages up until 2022 (framed in Fig. 4b), after which no other ST29-cl3 isolate was identified.

**Table 1.** Bayesian inference of key evolutionary parameters after BEAST2 analysis for RMP-ST21-cl5 and RMP-ST29-cl3

Clonal lineage	No. of isolates	TempEST correlation coefficient	TempEST *R*^2^	Selected model combination	tMRCA (95% HPD interval)	Substitution rate (substitution/site/year) (95% HPD interval)
RMP-ST21-cl5	37	0.59	0.34	(GTR+Γ4)+ strict clock+coalescent constant population	2003.176 (1996.764; 2008.6)	4.496×10^−7^ (3.182; 5.856)×10^−7^
RMP-ST29-cl3	33	0.74	0.54	(GTR+Γ4)+strict clock+coalescent Bayesian skyline	2017.372 (2016.438; 2017.91)	4.136×10^−7^ (2.387; 5.904)×10^−7^

## Discussion

STEC O26:H11 has globally emerged as a major HUS-inducing pathogen along with the historically prevalent O157:H7 serotype [8]. The global population of *E. coli* O26:H11 displays a high level of genetic diversity with two main STs, ST21 and ST29, further subdividing into four and two broad clades, respectively [50]. Previous studies showed that these clades are associated with different combinations of key genetic features carried by a large pO157-like virulence plasmid, pO26, including *ehxA* encoding enterohemolysin, *katP* encoding a catalase peroxidase, *espP* encoding a serine protease, *etpD* encoding a type III secretion system effector and *toxB* encoding an adherence-enhancing protein [50,54]. Over the last decades, several *stx2*-positive O26:H11 clones have emerged, including the new European clone which appeared in Germany in the 90s and subsequently spread across Europe [55,56] and the more recent *stx2d*-carrying new French clone described by Delannoy *et al*. [57]. However, *stx2*-carrying STEC O26:H11 has been shown to be diverse and to have emerged on multiple occasions among all STs and clonal lineages [50,58].

Based on whole-genome sequences of 130 *stx2*-positive STEC O26:H11 strains from bovine RMPs and from human infections, our study aimed at exploring the ecology of this pathogen and the interplay between these two reservoirs through genomic investigations of a fraction of their accessory genomes and their phylogenetic relationships.

The accessory genome offers valuable insight into bacterial ecology by revealing how strains adapt to distinct environmental niches and often harbours genetic elements responsible for virulence, such as pathogenicity islands, toxins and adhesion factors, which are critical for host colonization and disease progression [59,61]. Hence, to compare VAG-ARG-PR profiles between RMP and human isolates, we used an approach previously implemented by Michelacci and colleagues [32] on STEC O26:H11 human isolates only from Italy. Accessory gene patterns associated with either the human compartment or the RMP compartment were studied with an HCPC analysis based on the detection of virulence genes, AMR genes and PRs. This analysis revealed eight HCPC profiles with variations in accessory genetic features within the whole strain collection. For the most part, we found similar results to previous papers [32,50, 53, 55] where strains could be classified in the different HCPC profile established on different combinations of pO26-associated genetic determinants. For strains lacking the pO26 genetic markers (HCPC profiles 1, 2 and 3), we observed a differential partition of isolates from human source (grouped in HCPC clusters 1 and 2) and RMP (exclusively in HCPC cluster 3). Here, clinical isolates were positively associated with the presence of the *stx2d* gene subtype, rather than the *stx2a* subtype found in all RMP isolates, and with the presence of the *yghJ* gene encoding a type II secretion system metalloprotease involved in the human gastrointestinal tract colonization and commonly found in enterotoxigenic *E. coli* [62]. On the other hand, HCPC profile 5 comprised both human and RMP strains which carried the *stx2a* subtype, a combination of pO26 virulence genes (*ehxA*+/*katP*-/*espP*-/*etpD*+/*toxB*-) characteristic of the new European clone [55] and additional VAGs such as *iucC* and *iutA* involved in the aerobactin system, which is a high-affinity iron acquisition pathway found in avian pathogenic *E. coli* and extraintestinal pathogenic *E. coli* (ExPEC) [63,65]. Similar virulence features were evidenced in O26:H11 by the STEC-EURL in a previous study [32,66], reporting O26:H11 strains that carried the *iucC* and *iutA* genes on a partial pR444-like plasmid known to be frequently carried by highly pathogenic ST301 O80:H2 STEC-ExPEC strains [67]. Finally, HCPC profile 8 grouped the most isolates, also from both human and food origin, and displayed a nearly complete combination of pO26 genetic markers (*ehxA*+/*katP*+/*espP*+/*etpD*-/*toxB*-) with a set of ARGs. Also, HCPC clusters 6 and 8, comprising 76 isolates (76/130, 58.5%), were positively associated with the presence of multiple AMR genes including *aph3_Ib*, *aph6_Id*, *strA*, *strB*, *sul2* and *tetA/B* mediating resistance to aminoglycosides, sulphonamides and tetracyclines. Such observations are in line with previous findings for STEC O26 in cattle and humans [32,68,70] but also with resistance patterns reported by the French national surveillance programme for AMR in animal pathogens, the Resapath network [71]. The high observed prevalence of such genetic determinants in our bovine RMP strains aligns with the patterns of antimicrobial use in the bovine primary production sector where tetracyclines, aminoglycosides and sulphonamides are the first, third and fifth most prescribed antimicrobial classes [72,73].

The population structure of *stx2*-carrying O26:H11 STEC strains from the RMPs was further investigated, along with their clonal relationship with human isolates, through a core genome-based phylogenomic analysis of the whole strain collection. Genomes from this study were distributed across two broad clades, with clade A comprising ST29 strains and clade B mostly comprising ST21 strains and a small ST29 lineage. These two clades further subdivided into nine clonal lineages including two major lineages, ST29-cl3 from clade A and ST21-cl5 from clade B, that were present in the bovine raw milk production sector. Our analysis demonstrated similar clustering patterns of isolates based on their core genome and on the set of selected accessory genes with conserved accessory genome profiles within each clonal lineage. Indeed, ST21-cl5 mixed isolates from different host sources but with highly similar accessory gene profiles, except for a set of RMP isolates only displaying HCPC profile 6 instead of HCPC profile 8. RMP isolates from ST21-cl5 shared the same genetic backbone as human strains with some pairwise genetic distances of only 10 SNPs or less and relatively close isolation dates between two strains. The HCPC analysis results, taken together with the phylogenetic analysis of this particular STEC population, could nonetheless indicate a common source reservoir for ST21-cl5 strains found in bovine RMP and humans in France. However, in the absence of tangible epidemiological and contextual evidence supporting STEC transmission events from RMP to human, no conclusion can be drawn regarding source attribution of clinical isolates from this study [74,75]. Conversely, ST29-cl3, almost exclusively composed of RMP strains, corresponded to HCPC profile 2 and clustered separately from ST29-cl1 and ST29-cl2 which grouped clinical isolates only that displayed the HCPC profile 3. In this case, such clustering patterns support the hypothesis of a different transmission pathway than RMP for human infections with ST29-cl1 and cl2 strains. The imperfect correspondence between the core-genome-based analysis and the accessory gene profiles (VAG-AGR-PR) of the strains is consistent with findings by [32]. Indeed, we also observed a difference in conservation of accessory genetic features between lineages, with ST21 isolates displaying a more dynamic accessory genome compared to the high conservation seen in ST29 strains and suggesting that the ST21 population is particularly prone to the acquisition and loss of mobile genetic elements, leading to a rupture in the correspondence with the core-genome phylogeny. Our results thus highlight the differential role of the French bovine RMP sector depending on the considered clonal lineage. Also, given its economic and territorial organization, the French bovine raw milk production sector is a good candidate for phylogenomic studies of related foodborne pathogens as the vast majority of French RMPs fall under PDO [19,20]. These products are subjected to stringent specifications and regulations regarding their area of production, in which case a specific RMP must be produced with raw milk originating from dairy farms located in a defined area. Hence, our study not only provides a valuable overview of the pathogenic potentials and genetic diversity of *stx2*-carrying STEC O26:H11 found in RMP but also provides, admittedly partial, information regarding the circulating clones within the dairy cattle reservoir of relevant raw milk production regions.

Estimation of the tMRCA of these two major lineages provided evidence of relatively recent introduction in the French RMP sector around March 2003 [2003.176, 95% HPD interval (1996.764; 2008.6)] and May 2017 [2017.372, 95% HPD interval (2016.438; 2017.91)] for ST21-cl5 and ST29-cl3, respectively. Bayesian inference of a timed phylogeny for ST21-cl5 also revealed the existence of a geographically restricted persisting clone which appeared around 2015 in the Auvergne–Rhône–Alpes region and which was still detected up until the end of the study period (Fig. 4a). Interestingly, the timeline of appearance of this clone is consistent with the occurrence, a few years later, of two paediatric HUS outbreaks linked to the consumption of under PDO raw milk cheeses from this region and with similar outbreak isolates [15,16]. Besides this clone, other representative isolates of ST21-cl5 have broadly spread across multiple bovine raw milk-producing regions including Auvergne–Rhône–Alpes, Bourgogne–Franche–Comté, Normandie and Provence–Alpes–Côte-d'Azur. The other major ST29-cl3 found in RMP seems to have appeared even more recently in the Bourgogne–Franche–Comté region and was seldom detected outside of this region. Since mid-2022, no new ST29-cl3 strain was found in RMP, and only ST21-cl5 strains were found in this region from this date to the end of the study period, suggesting potential clonal turnover with a replacement of the ST29-cl3 lineage by the ST21-cl5 clonal lineage displaying higher pathogenic potential. For both lineages, the estimated substitution rates, 4,496×10^−7^ substitution/site/year for ST21-cl5 and 4,136×10^−7^ for ST29-cl3, were consistent with previous estimates for *E. coli* O26 [76] and other STEC serogroup [49,77, 78].

Several limitations should be considered regarding the initial strain collection available for this study. Indeed, the potential non-detection of other circulating clones within the RMP sector in France cannot be excluded. This limitation is particularly relevant given the absence of isolates from the Occitanie region, which represents ~10% of RMP production in France and given the limited quantity and diversity of isolates obtained from regions with lower raw milk production levels. To address these limitations, future research should prioritize the collection of strains from underrepresented regions to enable a more comprehensive phylogenomic analysis.

Finally, our study was limited to the human and the bovine RMP reservoirs and thus does not account for other known and unknown potential transmission sources. Indeed, it revealed a great diversity of *stx2*-positive O26:H11 strains found RMP with some circulating clonal lineages showing high pathogenic potentials and suggested that not all clinical isolates shared a common ecological niche with RMP isolates, confirming that other transmission pathways for human infection exist. Although the cross-sectoral genome matching process between HUS cases and isolates from other reservoirs is not as straightforward for STEC as for other foodborne pathogens, cross-reservoir genomic monitoring of STEC populations could provide a solid base for the early detection of emerging pathogenic clone and for better source attribution in the context of outbreak investigations [79,81].

In conclusion, a *stx2*-carrying O26:H11 clonal lineage, here denominated ST21-cl5, appeared in France circa 2003 and was found to have moderately diversified and become predominant in the raw milk production sector in multiple regions. ST21-cl5 isolates from the RMP sector displayed a high pathogenic potential as they clustered with HUS-associated isolates both based on their VAG-ARG-PR profiles and their core genomes. Such observations suggest that human and RMP ST21-cl5 isolates evolved under similar selective pressures and share a common ecological niche, probably the cattle reservoir, and that isolates from this lineage are highly adapted to this reservoir, allowing them to persist and continuously spread along the food chain. On the other hand, our work also suggests that O26:H11 *stx2d*-carrying STEC isolates, falling into the ST29-cl1 and ST29-cl2 clonal lineages, responsible for human infections might not stem from the raw milk production sector. Indeed, no *stx2d*-carrying strain was found among RMP isolates, and ST29 RMP strains displayed distinct VAG-ARG-PR profiles and genetic backbone compared with human ST29 *stx2d*-carrying isolates (commonly designated as the new French clone).

## Supplementary material

10.1099/mgen.0.001647 Uncited Supplementary Material 1.

10.1099/mgen.0.001647 Uncited Supplementary Material 2.

## Abbreviations

AMR: antimicrobial resistance
ARG: antimicrobial resistance gene
BEAST2: Bayesian Evolutionary Analysis Sampling Trees 2
cgMLST: core genome MLST
ExPEC: extra-intestinal pathogenic *Escherichia coli*
FNRL: French National Reference Laboratory
GTR: general time reversible
HCPC: hierarchical clustering on principal component
HPD interval: highest posterior density interval
HUS: haemolytic uraemic syndrome
iTOL: Interactive Tree of Life
MCA: multiple correspondence analysis
MLST: multilocus sequence type
MSA: multiple sequence alignment
PDO: protection of designated origin
PR: plasmid replicon
RMP: raw milk product
ST: sequence type
STEC: Shiga-toxin producing *Escherichia coli*
tMRCA: time of the most recent common ancestor
VAG: virulence-associated gene

## References

[1] Joseph A, Cointe A, Mariani Kurkdjian P, Rafat C, Hertig A (2020). Shiga toxin-associated hemolytic uremic syndrome: a narrative review. Toxins.

[2] Krüger A, Lucchesi PMA (2015). Shiga toxins and stx phages: highly diverse entities. Microbiology.

[3] Scheutz F, Teel LD, Beutin L, Piérard D, Buvens G (2012). Multicenter evaluation of a sequence-based protocol for subtyping Shiga toxins and standardizing Stx nomenclature. J Clin Microbiol.

[4] ANSES (2023). Avis de l’Agence nationale de sécurité sanitaire de l’alimentation, de l’environnement et du travail relatif à la définition des souches pathogènes d’Escherichia coli productrices de shigatoxines.

[5] ANSES (2011). Avis de l’Agence nationale de la sécurité sanitaire de l’alimentation, de l’environnement et du travail relatif à la révision de la définition des *E. coli* entéro-hémorragiques (EHEC) majeurs typiques, à l’appréciation quantitative des risques liés à ces bactéries à différentes étapes de la chaîne alimentaire, selon les différents modes de consommation des steaks hachés, et à la prise en compte du danger lié aux *E. coli* entéro-pathogènes (EPEC) dans les aliments.

[6] Santé Publique France (2024). Santé publique france. surveillance du syndrome hémolytique et urémique typique chez l’enfant de moins de 15 ans en france en 2023.

[7] CNR *E. coli*, Shigella et Salmonella. Rapport d’activité annuel 2024: Année d’exercice 2023.

[8] European Food Safety Authority (EFSA), European Centre for Disease Prevention and Control (ECDC) (2024). The European Union one health 2023 zoonoses report. EFSA J.

[9] Boerlin P, McEwen SA, Boerlin-Petzold F, Wilson JB, Johnson RP (1999). Associations between virulence factors of Shiga toxin-producing *Escherichia coli* and disease in humans. J Clin Microbiol.

[10] Koutsoumanis K, Allende A, Alvarez‐Ordóñez A, Bover‐Cid S, Chemaly M (2020). Pathogenicity assessment of Shiga toxin‐producing *Escherichia coli* (STEC) and the public health risk posed by contamination of food with STEC. *EFS2*.

[11] Fuller CA, Pellino CA, Flagler MJ, Strasser JE, Weiss AA (2011). Shiga toxin subtypes display dramatic differences in potency. Infect Immun.

[12] FAO, WHO (2022). Control Measures for Shiga Toxin-Producing Escherichia Coli (STEC) Associated with Meat and Dairy Products.

[13] Bruyand M, Mariani-Kurkdjian P, Le Hello S, King L-A, Van Cauteren D (2019). Paediatric haemolytic uraemic syndrome related to Shiga toxin-producing *Escherichia coli*, an overview of 10 years of surveillance in France, 2007 to 2016. Euro Surveill.

[14] Ganet S, Felix S, Werlen S, Lusurier L, Faure-Bondat A (2024). Shiga-toxin *Escherichia coli* (STEC) isolated from foods and environment between 2018 and 2022 by the French national reference laboratory of *E. coli*.

[15] Santé Publique France (2019). Epidémie de SHU pédiatrique à *E. coli* O26 en France métropolitaine en lien avec la consommation de fromages Saint Marcellin et Saint Félicien: point de situation au 28 mai 2019. Santé Publique Fr 2019. https://www.santepubliquefrance.fr/les-actualites/2019/epidemie-de-shu-pediatrique-a-e.-coli-o26-en-france-metropolitaine-en-lien-avec-la-consommation-de-fromages-saint-marcellin-et-saint-felicien-poi.

[16] Jones G, Valk H (2020). Épidémie d’infections à *E.coli* O26 producteurs de shiga-toxine liés à la consommation de reblochon au lait cru, France, Mars-Mai 2018.

[17] Minary K, Tanne C, Kwon T, Faudeux C, Clave S (2022). Outbreak of hemolytic uremic syndrome with unusually severe clinical presentation caused by Shiga toxin-producing *Escherichia coli* O26:H11 in France. *Archives de Pédiatrie*.

[18] Ministère de la Santé (2023). Rappel de fromages à pâte pressée non cuites à base de lait cru. https://sante.gouv.fr/actualites/presse/communiques-de-presse/article/les-autorites-rappellent-les-recommandations-sanitaires-dans-le-cadre-du.

[19] (2024). CNAOL, INAO. Chiffres clés 2023 des produits sous signes de la qualité et de l’origine - Produits laitiers AOP et IGP.

[20] CNIEL, ANICAP, CNAOL (2022). Les chiffres clés des fromages au lait cru. https://www.fromagesaulaitcru.fr.

[21] Bogaerts B, Nouws S, Verhaegen B, Denayer S, Van Braekel J (2021). Validation strategy of a bioinformatics whole genome sequencing workflow for Shiga toxin-producing *Escherichia coli* using a reference collection extensively characterized with conventional methods. Microbial Genomics.

[22] Andrews S (2010). FastQC: a quality control tool for high throughput sequence data. http://www.bioinformatics.babraham.ac.uk/projects/fastqc.

[23] Bolger AM, Lohse M, Usadel B (2014). Trimmomatic: a flexible trimmer for Illumina sequence data. Bioinformatics.

[24] Bankevich A, Nurk S, Antipov D, Gurevich AA, Dvorkin M (2012). SPAdes: a new genome assembly algorithm and its applications to single-cell sequencing. J Comput Biol.

[25] Joensen KG, Tetzschner AMM, Iguchi A, Aarestrup FM, Scheutz F (2015). Rapid and easy in silico serotyping of *Escherichia coli* isolates by use of whole-genome sequencing data. J Clin Microbiol.

[26] Joensen KG, Scheutz F, Lund O, Hasman H, Kaas RS (2014). Real-time whole-genome sequencing for routine typing, surveillance, and outbreak detection of verotoxigenic *Escherichia coli*. J Clin Microbiol.

[27] Malberg Tetzschner AM, Johnson JR, Johnston BD, Lund O, Scheutz F (2020). *In Silico* genotyping of *Escherichia coli* isolates for extraintestinal virulence genes by use of whole-genome sequencing data. J Clin Microbiol.

[28] Carattoli A, Hasman H (2020). PlasmidFinder and In Silico pMLST: identification and typing of plasmid replicons in whole-genome sequencing (WGS). Methods Mol Biol.

[29] Bortolaia V, Kaas RS, Ruppe E, Roberts MC, Schwarz S (2020). ResFinder 4.0 for predictions of phenotypes from genotypes. J Antimicrob Chemother.

[30] Wirth T, Falush D, Lan R, Colles F, Mensa P (2006). Sex and virulence in *Escherichia coli*: an evolutionary perspective. Mol Microbiol.

[31] Camacho C, Coulouris G, Avagyan V, Ma N, Papadopoulos J (2009). BLAST+: architecture and applications. BMC Bioinformatics.

[32] Michelacci V, Montalbano Di Filippo M, Gigliucci F, Arancia S, Chiani P (2022). Population analysis of O26 shiga toxin-producing *Escherichia coli* causing hemolytic uremic syndrome in Italy, 1989-2020, through whole genome sequencing. Front Cell Infect Microbiol.

[33] Husson F, Josse J, Pagès J (2010). Principal component methods - hierarchical clustering - partitional clustering: why would we need to choose for visualizing data. Tech Rep – Agrocampus.

[34] R Core Team R: a language and environment for statistical computing.

[35] Lê S, Josse J, Husson F (2008). FactoMineR: an r package for multivariate analysis. J Stat Softw.

[36] Lebart L, Morineau A, Piron M (1997). Statistique Exploratoire Multidimensionnelle.

[37] Silva M, Machado MP, Silva DN, Rossi M, Moran-Gilad J (2018). chewBBACA: a complete suite for gene-by-gene schema creation and strain identification. Microbial Genomics.

[38] Seemann T (2015). Snippy: rapid haploid variant calling and core genome alignment.

[39] Croucher NJ, Page AJ, Connor TR, Delaney AJ, Keane JA (2015). Rapid phylogenetic analysis of large samples of recombinant bacterial whole genome sequences using Gubbins. Nucleic Acids Res.

[40] Nguyen L-T, Schmidt HA, von Haeseler A, Minh BQ (2015). IQ-TREE: a fast and effective stochastic algorithm for estimating maximum-likelihood phylogenies. Mol Biol Evol.

[41] Kalyaanamoorthy S, Minh BQ, Wong TKF, von Haeseler A, Jermiin LS (2017). ModelFinder: fast model selection for accurate phylogenetic estimates. Nat Methods.

[42] Letunic I, Bork P (2021). Interactive Tree Of Life (iTOL) v5: an online tool for phylogenetic tree display and annotation. Nucleic Acids Res.

[43] Seemann T (2019). SNP.dist: pairwise SNP distance matrix from a FASTA sequence alignment.

[44] Rambaut A, Lam TT, Max Carvalho L, Pybus OG (2016). Exploring the temporal structure of heterochronous sequences using TempEst (formerly Path-O-Gen). Virus Evol.

[45] Bouckaert R, Vaughan TG, Barido-Sottani J, Duchêne S, Fourment M (2019). BEAST 2.5: an advanced software platform for Bayesian evolutionary analysis. PLoS Comput Biol.

[46] Russel PM, Brewer BJ, Klaere S, Bouckaert RR (2019). Model selection and parameter inference in phylogenetics using nested sampling. Syst Biol.

[47] Rambaut A, Drummond AJ, Xie D, Baele G, Suchard MA (2018). Posterior summarization in Bayesian phylogenetics using tracer 1.7. Syst Biol.

[48] Duchêne S, Holt KE, Weill F-X, Le Hello S, Hawkey J (2016). Genome-scale rates of evolutionary change in bacteria. Microbial Genomics.

[49] Wang LYR, Jokinen CC, Laing CR, Johnson RP, Ziebell K (2020). Assessing the genomic relatedness and evolutionary rates of persistent verotoxigenic *Escherichia coli* serotypes within a closed beef herd in Canada. Microbial Genomics.

[50] Ogura Y, Gotoh Y, Itoh T, Sato MP, Seto K (2017). Population structure of *Escherichia coli* O26 : H11 with recent and repeated stx2 acquisition in multiple lineages. Microbial Genomics.

[51] Jenkins C, Evans J, Chart H, Willshaw GA, Frankel G (2007). *Escherichia coli* serogroup O26 ? A new look at an old adversary. J Appl Microbiol.

[52] Zweifel C, Cernela N, Stephan R (2013). Detection of the emerging Shiga toxin-producing *Escherichia coli* O26:H11/H- sequence type 29 (ST29) clone in human patients and healthy cattle in Switzerland. Appl Environ Microbiol.

[53] Ishijima N, Lee K-I, Kuwahara T, Nakayama-Imaohji H, Yoneda S (2017). Identification of a new virulent clade in enterohemorrhagic *Escherichia coli* O26:H11/H- sequence type 29. Sci Rep.

[54] Long J, Geng J, Xu Y, Jin Y, Yang H (2022). Large-scale phylogenetic analysis reveals a new genetic clade among *Escherichia coli* O26 strains. Microbiol Spectr.

[55] Bielaszewska M, Mellmann A, Bletz S, Zhang W, Köck R (2013). Enterohemorrhagic *Escherichia coli* O26:H11/H-: a new virulent clone emerges in Europe. Clin Infect Dis.

[56] Karnisova L, Marejkova M, Hrbackova H, Mellmann A, Karch H (2018). Attack of the clones: whole genome-based characterization of two closely related enterohemorrhagic *Escherichia coli* O26 epidemic lineages. BMC Genom.

[57] Delannoy S, Mariani-Kurkdjian P, Bonacorsi S, Liguori S, Fach P (2015). Characteristics of emerging human-pathogenic *Escherichia coli* O26:H11 strains isolated in France between 2010 and 2013 and carrying the stx2d gene only. J Clin Microbiol.

[58] Bonanno L, Loukiadis E, Mariani-Kurkdjian P, Oswald E, Garnier L (2015). Diversity of shiga toxin-producing *Escherichia coli* (STEC) O26:H11 strains examined via stx subtypes and insertion sites of Stx and EspK bacteriophages. Appl Environ Microbiol.

[59] Foster-Nyarko E, Pallen MJ (2022). The microbial ecology of *Escherichia coli* in the vertebrate gut. FEMS Microbiol Rev.

[60] Nielsen KL, Stegger M, Kiil K, Godfrey PA, Feldgarden M (2017). Whole-genome comparison of urinary pathogenic *Escherichia coli* and faecal isolates of UTI patients and healthy controls. Int J Med Microbiol.

[61] McNally A, Oren Y, Kelly D, Pascoe B, Dunn S (2016). Combined analysis of variation in core, accessory and regulatory genome regions provides a super-resolution view into the evolution of bacterial populations. PLOS Genet.

[62] Tapader R, Bose D, Pal A (2017). YghJ, the secreted metalloprotease of pathogenic *E. coli* induces hemorrhagic fluid accumulation in mouse ileal loop. Microb Pathog.

[63] de Lorenzo V, Neilands JB (1986). Characterization of iucA and iucC genes of the aerobactin system of plasmid ColV-K30 in *Escherichia coli*. J Bacteriol.

[64] Ling J, Pan H, Gao Q, Xiong L, Zhou Y (2013). Aerobactin synthesis genes iucA and iucC contribute to the pathogenicity of avian pathogenic *Escherichia coli* O2 strain E058. PLoS ONE.

[65] Ikeda M, Kobayashi T, Fujimoto F, Okada Y, Higurashi Y (2021). The prevalence of the iutA and ibeA genes in *Escherichia coli* isolates from severe and non-severe patients with bacteremic acute biliary tract infection is significantly different. Gut Pathog.

[66] Gigliucci F, van Hoek AHAM, Chiani P, Knijn A, Minelli F (2021). Genomic characterization of hlyF-positive shiga toxin-producing *Escherichia coli*, Italy and the Netherlands, 2000-2019. Emerg Infect Dis.

[67] Cointe A, Birgy A, Mariani-Kurkdjian P, Liguori S, Courroux C (2018). Emerging multidrug-resistant hybrid pathotype shiga toxin-producing *Escherichia coli* O80 and related strains of clonal complex 165, Europe. *Emerg Infect Dis*.

[68] Lee JH (2009). Antimicrobial resistance of *Escherichia coli* O26 and O111 isolates from cattle and their characteristics. Vet Microbiol.

[69] Boireau C, Morignat É, Cazeau G, Jarrige N, Jouy É (2018). Antimicrobial resistance trends in *Escherichia coli* isolated from diseased food-producing animals in France: a 14-year period time-series study. Zoonoses Public Health.

[70] Hoyle DV, Wee BA, Macleod K, Chase-Topping ME, Bease AG (2023). Phylogenetic relationship and virulence composition of *Escherichia coli* O26:H11 cattle and human strain collections in Scotland; 2002-2020. Front Microbiol.

[71] ANSES Résapath - Réseau d’épidémiosurveillance de l’antibiorésistance des bactéries pathogènes animales: Bilan 2023.

[72] ANSES-ANMV (2024). Médicaments antimicrobiens chez l’animal - surveillance des ventes et des utilisations pour l’année 2023.

[73] Gay E (2012). Utilisation des antibiotiques chez les ruminants domestiques en france: résultats d’enquêtes de pratiques auprès d’éleveurs et de vétérinaires.

[74] Pightling AW, Pettengill JB, Luo Y, Baugher JD, Rand H (2018). Interpreting whole-genome sequence analyses of foodborne bacteria for regulatory applications and outbreak investigations. Front Microbiol.

[75] Nouws S, Verhaegen B, Denayer S, Crombé F, Piérard D (2023). Transforming Shiga toxin-producing *Escherichia coli* surveillance through whole genome sequencing in food safety practices. Front Microbiol.

[76] Browne AS, Biggs PJ, Wilkinson DA, Cookson AL, Midwinter AC (2019). Use of Genomics to Investigate Historical Importation of Shiga Toxin-Producing *Escherichia coli* Serogroup O26 and Nontoxigenic Variants into New Zealand. *Emerg Infect Dis*.

[77] Weinroth MD, Clawson ML, Arthur TM, Wells JE, Brichta-Harhay DM (2022). Rates of evolutionary change of resident *Escherichia coli* O157:H7 differ within the same ecological niche. BMC Genomics.

[78] Dallman TJ, Ashton PM, Byrne L, Perry NT, Petrovska L (2015). Applying phylogenomics to understand the emergence of Shiga-toxin-producing *Escherichia coli* O157:H7 strains causing severe human disease in the UK. Microb Genomics.

[79] Baert L, McClure P, Winkler A, Karn J, Bouwknegt M (2021). Guidance document on the use of whole genome sequencing (WGS) for source tracking from a food industry perspective. Food Control.

[80] Moran-Gilad J (2017). Whole genome sequencing (WGS) for food-borne pathogen surveillance and control - taking the pulse. Euro Surveill.

[81] Friesema IH, van der Voort M, Wit B, van Hoek AH, van den Beld MJ (2024). Cross-sectoral genomic surveillance reveals a lack of insight in sources of human infections with Shiga toxin-producing *Escherichia coli*, the Netherlands, 2017 to 2023. Euro Surveill.

