# Supplementary material for: Pathogenic potential and phylogenomic analysis of stx2-carrying O26:H11 Shiga toxin-producing Escherichia coli isolated from dairy products in France (2014–2024)
Source: Microb Genom. 2026 Feb 19;12(2):001647. doi: 10.1099/mgen.0.001647 (PMC12935931; doi:10.1099/mgen.0.001647)
Supplement: Uncited Supplementary Material 1. [file mgen-12-01647-s001.pdf]

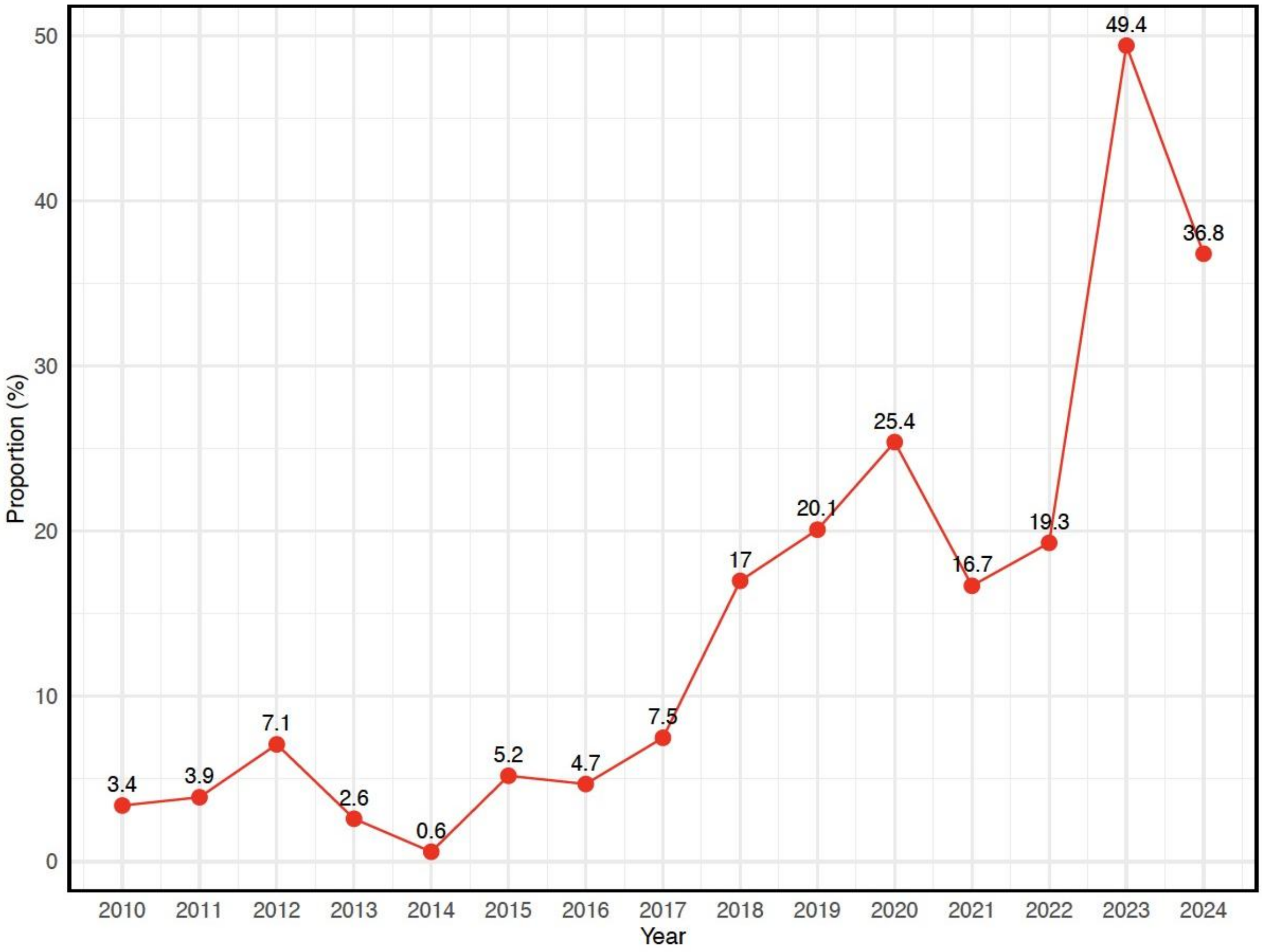

Figure S1 – Proportion of stx2-carrying strains among all O26 :H11 strains isolated by the French national reference laboratory between 2010 and 2014.

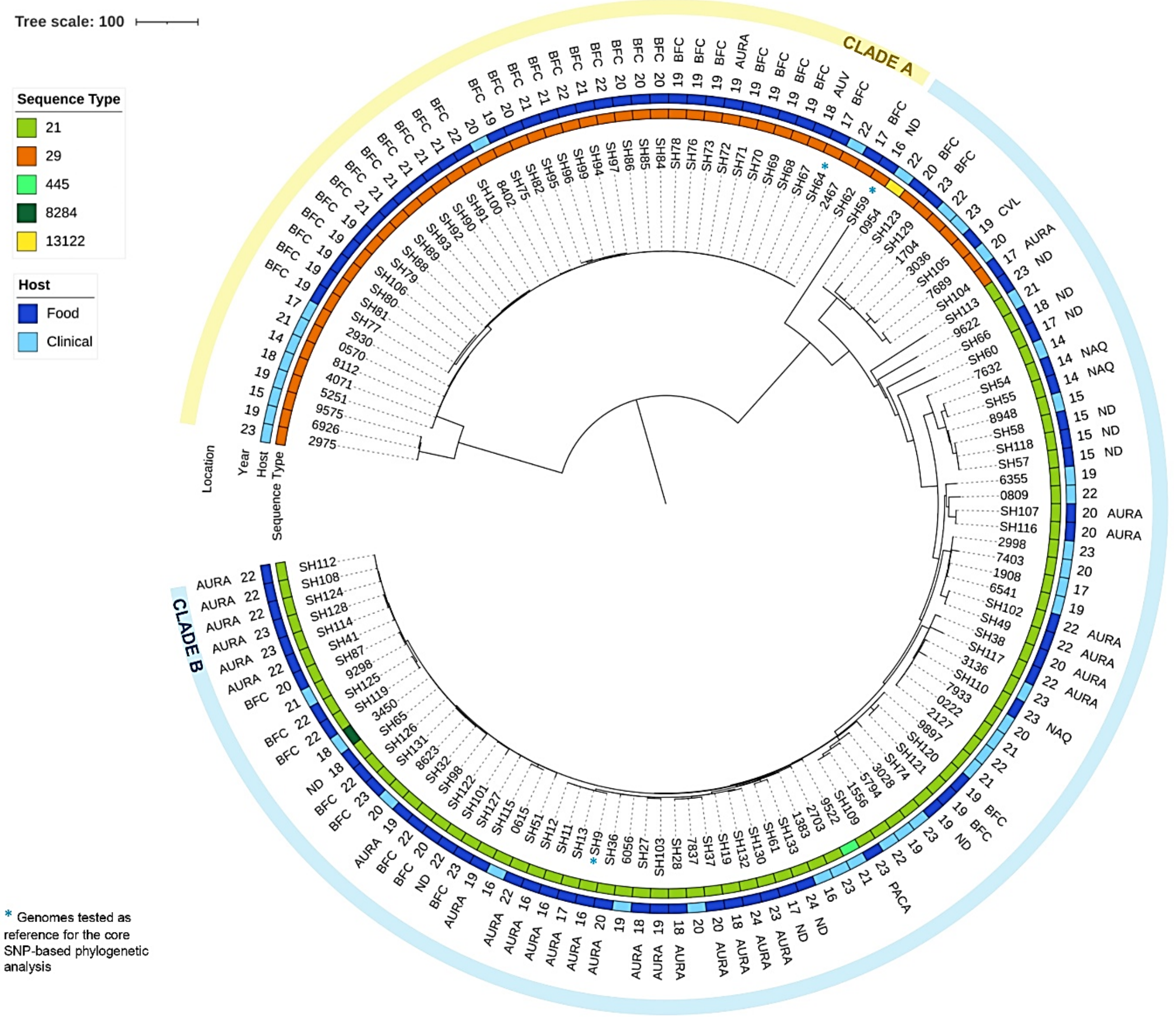

Figure S2 – Phylogenetic tree based on cgMLST analysis (E. coli INNUENDO scheme). Metadata associated with location, year of isolation (e.g. 22 means 2022), host type and sequence type are represented by colored rings. Abbreviations for the regions of production for raw milk product and the regions of origin for clinical cases are the following: AURA, Auvergne-Rhône-Alpes; BFC, Bourgogne-Franche-Comté; BR, Bretagne, CVL, Centre-Val-de-Loire; GE, Grand-Est; HDF, Haut-de-France; IDF, Ile-de-France; ND, Normandie; NAQ, Nouvelle-Aquitaine, OCC, Occitanie; PDL, Pays-de-la-Loire ; PACA, Provence-Alpes-Côte-d'Azur. Genomes subsequently tested as the reference genome for core SNP-based phylogenetic analysis are indicated with a blue asterisk.

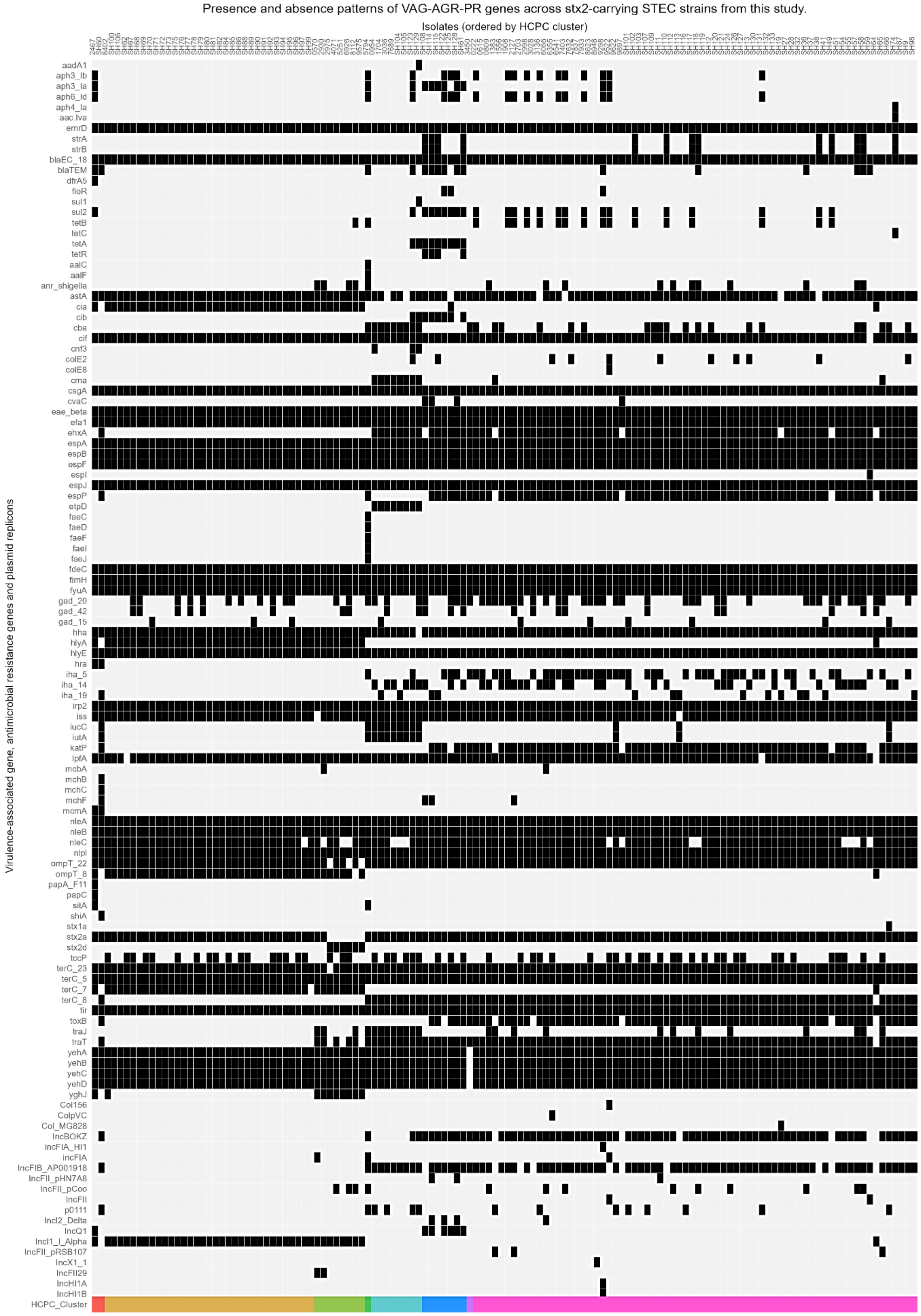

Figure S3 - Presence/Absence patterns of a selection of accessory genes (VAG, AGR, PR) in stx2-carrying STEC isolates included in this study.

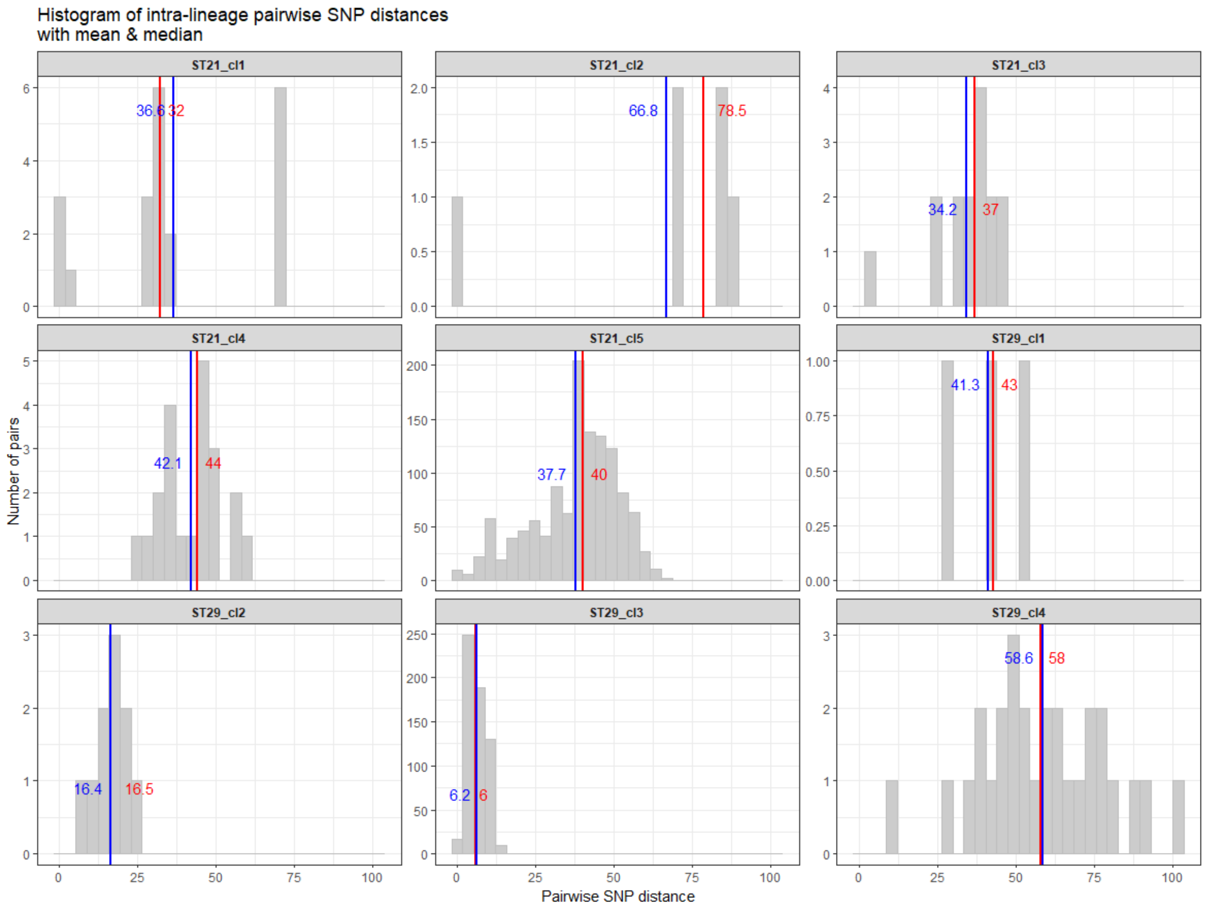

Figure S4 - Histogram of intra-lineage pairwise core-SNP distances with mean (blue) and median (red) pairwise SNP distances.
